# B-Chromosome Ribosomal DNA Is Functional in the Grasshopper *Eyprepocnemis plorans*

**DOI:** 10.1371/journal.pone.0036600

**Published:** 2012-05-03

**Authors:** Mercedes Ruiz-Estévez, M Dolores López-León, Josefa Cabrero, Juan Pedro M. Camacho

**Affiliations:** Departamento de Genética, Universidad de Granada, Granada, Spain; Florida State University, United States of America

## Abstract

B-chromosomes are frequently argued to be genetically inert elements, but activity for some particular genes has been reported, especially for ribosomal RNA (rRNA) genes whose expression can easily be detected at the cytological level by the visualization of their phenotypic expression, i.e., the nucleolus. The B_24_ chromosome in the grasshopper *Eyprepocnemis plorans* frequently shows a nucleolus attached to it during meiotic prophase I. Here we show the presence of rRNA transcripts that unequivocally came from the B_24_ chromosome. To detect these transcripts, we designed primers specifically anchoring at the ITS-2 region, so that the reverse primer was complementary to the B chromosome DNA sequence including a differential adenine insertion being absent in the ITS2 of A chromosomes. PCR analysis carried out on genomic DNA showed amplification in B-carrying males but not in B-lacking ones. PCR analyses performed on complementary DNA showed amplification in about half of B-carrying males. Joint cytological and molecular analysis performed on 34 B-carrying males showed a close correspondence between the presence of B-specific transcripts and of nucleoli attached to the B chromosome. In addition, the molecular analysis revealed activity of the B chromosome rDNA in 10 out of the 13 B-carrying females analysed. Our results suggest that the nucleoli attached to B chromosomes are actively formed by expression of the rDNA carried by them, and not by recruitment of nucleolar materials formed in A chromosome nucleolar organizing regions. Therefore, B-chromosome rDNA in *E. plorans* is functional since it is actively transcribed to form the nucleolus attached to the B chromosome. This demonstrates that some heterochromatic B chromosomes can harbour functional genes.

## Introduction

B chromosomes constitute a bizarre part of the genomes in about 15% of eukaryote species, being dispensable and frequently harmful for carrier individuals, despite they sometimes reach high population frequencies thanks to conspicuous mechanisms for advantageous transmission (drive). The DNA contained into B chromosomes is a broad panoply of repetitive sequences, including satellite and microsatellite DNA, ribosomal DNA (rDNA) and mobile elements [1]. Recently, the presence of H3 and H4 histone genes has been shown in the B chromosomes of *Locusta migratoria* [2].

The kind of repetitive DNA most frequently found in B chromosomes is 45S rDNA [1], [3], [4]. It is located at chromosome sites named nucleolus organizer regions (NORs) consisting of tandemly repeated units composed of 18S, 5.8S and 28S rDNA, separated by two internal transcribed spacers (ITS1 and ITS2) and flanked by external transcribed spacers (ETS) and non-transcribed spacers (NTS) [5]. A cytologically visible phenotype for these genes is the nucleolus, a nuclear membrane-free compartment where ribosome components are synthesized [6]. It may easily be revealed in both interphase and meiotic cells by silver impregnation [7]. This technique specifically reveals the transcriptional machinery of RNA polimerase I, including the B23, nucleolin, UBF and ARN Pol I subunits proteins [8]–[10]. The size of the nucleolus is proportional to its biosynthetic activity [11]–[17].

B chromosomes were considered genetically inert for long, since experiments with tritiated uridine revealed its scarce incorporation into B chromosomes in the grasshoppers *Myrmeleotettix maculatus* and *Chorthippus parallelus* [18] and the mouse *Apodemus peninsulae* [19]. In maize, B chromosomes are basically inert [20]. However, indirect evidence for transcription from B chromosomes was obtained in the toad *Leiopelma hochstetteri* [21] and the mosquito *Simulium juxtacrenobium* [22]. Recently, evidence for a functional role of B chromosomes on female sex determination has been shown in cichlid fishes [23].

Some B chromosomes have been shown to carry a NOR being able to organize nucleoli. For instance, in the grasshopper *Dichroplus pratensis*, a B chromosome was frequently associated to a nucleolus during meiosis [24], and it was later shown, by fluorescent in situ hybridization (FISH), that it carries rDNA [25]. In many cases, however, the rDNA located on the B chromosomes is inactive, as found, for instance, in the black rat *Rattus rattus* [26] and the fish species *Metynnis maculatus* [27] and *Haplochromis obliquidens* [28]. Especially puzzling is the case of the plant *Brachychome dichromosomatica*, where large B chromosomes carrying rRNA genes are often associated with a nucleolus at mitotic prophase cells in root tips and in meiosis of pollen mother cells [29], but these genes do not silver stain and no transcripts were detected coming from them in leaves [30]. On the other hand, the rare nucleolar association of micro B chromosomes suggests inactivity of its 45S rDNA [31].

The first molecular evidence for gene activity on B chromosomes was found in the plant *Crepis capillaris* [32], specifically for rDNA. In the parasitic wasp *Trichogramma kaykai*, NOR activity of B chromosomes and presence of rRNA transcripts coming from the Bs has also been reported [33]. In rye, it has recently been shown the transcription of B-specific DNA sequences belonging to high-copy number families with similarity to mobile elements [34].

In the grasshopper *Eyprepocnemis plorans*, FISH has shown that most A chromosomes carry rDNA, although the highest amount is actually found in the B chromosomes [35]. However, in most cases, NOR activity is only observed in the three smallest autosomes (9, 10 and 11) and the X chromosome, but not in the B. Cabrero *et al.* [36] reported the presence of nucleoli associated to a B chromosome that had fused to the longest autosome, in strong contrast to non-fused Bs where such NOR activity had never been found. It was later shown that rDNA is one of the principal components of B chromosomes in this species [35], and we have recently found a natural population where the rDNA in the B_24_ chromosome is recurrently active, as deduced from the presence of nucleoli attached to B chromosomes [37], [38]. It was unknown, however, whether those B-attached nucleoli are formed *in situ* by expression of the B chromosome rDNA, or else by recruitment of nucleolar material coming from other nucleolar organizer regions (NORs), and therefore without expression of the B rDNA. The main objective of the present research was to test for these two alternatives by trying to detect rRNA transcripts that undoubtedly had come from the B chromosome rDNA. For this purpose, we used previous information provided by Teruel [39], who determined the DNA sequence of the ITS1 and ITS2 of rDNA coming from microdissected B_24_ chromosomes in *E. plorans*. A comparison with the corresponding DNA sequence in the A chromosomes, obtained from 0B individuals, revealed that most B-derived ITS2 sequences carried an inserted adenine which was absent in all A-derived sequences [39]. This difference could thus be used for identifying the rRNA transcripts coming from the B chromosome. We then developed a PCR-based molecular method to detect the B-rRNA transcripts in B-carrying males and, as a cytological control, analysed the presence of nucleoli attached to B chromosomes at diplotene. This molecular approach also demonstrated to be useful to find B-rRNA transcripts in females.

## Results

### Cytological analysis

**Figure 1:**
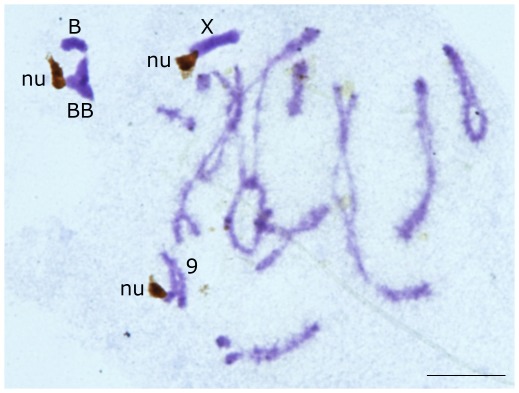
Nucleolus formation by B chromosomes. Silver stained diplotene cell showing nucleoli (nu) attached to a B bivalent (BB), the X chromosome and autosomal bivalent no. 9. Bar = 10 µ.

Table 1 shows the number of B chromosomes observed in the 67 individuals analysed. Silver impregnation of diplotene cells revealed the presence of nucleoli attached to B chromosomes in 14 out of the 29 B-carrying males collected in 2008 (Figure 1), but not in the 15 remaining males (Table 2). Therefore, 48.3% of B-carrying males showed NOR activity in the B chromosomes. This figure did not differ from that reported by Teruel *et al.* [38] in a sample from this same population collected in 2004, where 53% of 36 B-carrying males showed B-NOR activity (contingency χ^2^ = 0.02, df = 1, P = 0.89). This shows that the frequency of B-carrying males showing NOR activity in the B chromosomes has not significantly changed in these four years. As Table 2 shows, the likelihood of showing NOR activity in a B chromosome increased with B number, at least from 1–3 Bs, the exception being a single 4B male failing to show active Bs.

**Table 1:** Number of B chromosomes in the 67 individuals collected in the Torrox (Málaga, Spain) population of the grasshopper *Eyprepocnemis plorans*.

Number of Bs	♂♂ 2003	♂♂ 2004	♀♀ 2007	♂♂ 2008	Total
0	-	-	10	10	20
1	-	1	2	19	21
2	1	-	11	5	18
3	1	2	-	4	7
4	-	-	-	1	1
**Total**	2	3	23	39	67

**Table 2:** Frequency of B-carrying males from the 2008 sample showing NOR activity in the B chromosomes, as deduced from the presence of nucleoli attached to B chromosomes in diplotene cells.

Number of Bs	Males with inactive Bs	Males with active Bs	Total	% Males with active Bs
1	13	6	19	31.6
2	1	4	5	80
3	0	4	4	100
4	1	0	1	0
**Total**	15	14	29	48.3

In the 14 males showing B-NOR activity, a rather regular proportion of diplotene cells showed B-attached nucleoli: 23.3% in 1B, 23.7% in 2B and 26.9% in 3B males (Table 3), suggesting that it does not depend on the number of B chromosomes. The cytological analysis of the five B-carrying males collected in 2003 and 2004 showed nucleoli attached to Bs in all of them, since they had been selected from a larger sample previously analysed [39]. The average frequency of diplotene cells showing B-attached nucleoli in males showing B-NOR expression in the 2008 sample (24.7% of 284 cells analysed in 14 males), did not differ from the values previously observed in this same population (23.2% of 69 cells in 1999, 23.3% of 240 cells in 2003 and 19.5% of 380 cells in 2004) [37], [38] (contingency χ^2^ = 2.84, df = 3, P = 0.42).

**Table 3:** Proportion of diplotene cells showing nucleoli attached to B chromosomes in 14 B-carrying males collected in Torrox in 2008 and therefore showing NOR activity in the B chromosome.

		No. of cells showing			% B-NOR activity
Number of Bs	Male no.	B-NOR inactivity	B-NOR activity	Total	
1	54	10	10	20	50.0
	49	14	6	20	30.0
	58	16	4	20	20.0
	67	15	5	20	25.0
	52	19	1	20	5.0
	45	18	2	20	10.0
	**Total:**	92	28	120	23.3
2	74	18	2	20	10.0
	61	14	6	20	30.0
	63	15	5	20	25.0
	55	12	8	20	40.0
	**Total:**	59	21	80	23.7
3	62	15	9	24	37.5
	50	13	7	20	35.0
	42	17	3	20	15.0
	71	16	4	20	20.0
	**Total:**	61	23	84	26.9
**Total**		212	72	284	24.7

### Molecular analysis

In each individual, we performed PCR experiments with the ITSA and ITSB primers on both genomic (gDNA) and complementary (cDNA) DNA. The former served as a positive control for the presence of the B-specific ITS2 with the inserted adenine (ITS2_B), whereas the cDNA analysis tested the presence of rRNA transcripts carrying the B-specific ITS2.

**Figure 2:**
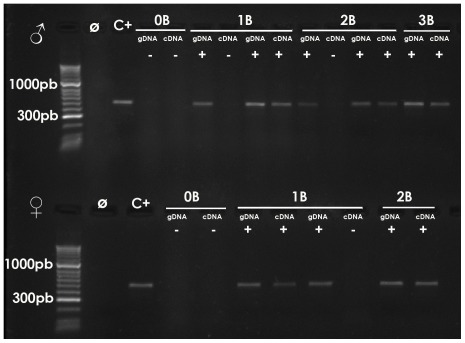
Amplification of the ITS2_B region with the ITSA and ITSB primers on genomic (gDNA) and complementary (cDNA) DNA from representative males with 0–3 B chromosomes (upper panel) and females with 0–2 B chromosomes (lower panel). Note the presence of PCR product on gDNA of B-carrying individuals but absence in the case of B-lacking ones. Also note the presence of PCR product on cDNA of only some B-carrying individuals. Ø = Negative control (with no DNA). C^+^ = Positive control (gDNA from 1B male).

The PCR experiments on gDNA showed amplification in all B-carrying males and females but not in any of the B-lacking ones (Figure 2). Therefore, this PCR reaction shows the appropriate specificity to reveal B chromosome (and ITS2_B) presence. Cloning and sequencing of the obtained band revealed that it corresponded to the expected ITS2_B region (Figure S1).

The PCR analysis on cDNA with the ITSA and ITSB primers in B-lacking individuals, as a negative control, revealed no amplification in both males and females (Figure 2), as expected from the results of the PCR experiments on gDNA. However, the molecular analysis of all B-carrying individuals (34 males and 13 females), showed that the ITS2_B transcript was present in the cDNA of 20 of these males (9 with 1B, 4 with 2B and 7 with 3B) but not in 14 of them (11 with 1B, 2 with 2B and 1 with 4B). Likewise, 10 B-carrying females (2 with 1B and 8 with 2B) showed the presence of the 484 bp ITS2_B transcript, whereas 3 females with 2B failed to show it. The frequency of B-carrying individuals showing ITS2_B transcripts was thus 58.8% in males and 76.9% in females.

The joint cytological and molecular analysis performed in 34 B-carrying males (also including five males collected in 2003 and 2004) revealed that 18 out of the 20 males showing ITS2_B transcripts also showed nucleoli attached to B chromosomes in diplotene cells, whereas the two remaining males (no. 43 and 72, both with 1B) failed to show evidence of B-rDNA expression at the cytological level. On the other hand, one male (no. 74) failed to show the presence of the ITS2_B transcript but showed nucleoli attached to Bs in two out of the 20 cells analysed (see Table 3). Finally, 13 males showed absence of B-rDNA expression at both cytological and molecular levels (Table 4). A contingency chi-square test performed to this 2×2 table showed a very strong association between the presence of nucleoli attached to the B chromosomes and the presence of the ITS2_B transcript (χ^2^_1_ = 19.69, P<0.0001, with Yates' correction).

**Table 4:** Joint cytological and molecular analysis of B chromosome NOR expression in 34 males of the grasshopper *E. plorans*.

	Nucleolus attached to the B chromosome	
Molecular detection of the ITS2_B transcript	+	−
+	18	2
−	1	13

## Discussion

A number of evidences have shown that B chromosomes in the grasshopper *Eyprepocnemis plorans* are sometimes attached to a nucleolus, suggesting the possibility of expression of their rDNA. In addition to previous evidences of NOR activity on B chromosomes [36]–[39], our present cytological analysis, by means of silver impregnation, in the 29 B-carrying males collected in this same population, in 2008, has shown that the frequency of males showing NOR activity in B chromosomes has not changed from 2004 to 2008, being close to 50%. In addition, the average proportion of diplotene cells showing B-NOR activity seemed to be rather stable (about 20%) from 1999 to 2008. This suggests that the phenotypic expression of the “active B-NOR” trait shows high temporal stability in this population. In contrast, expresivity of this trait is highly variable among individuals, i.e. 5–50% (see Table 3).

Although nucleoli are usually formed by expression of the rDNA contained in the NOR to which they are attached [6], in the case of non-standard genomic elements like parasitic B chromosomes, the possibility exists that B chromosomes could recruit nucleolar materials from other NORs without the need to expressing their own rDNA. Our present results show that it is possible to detect the presence of rRNA transcripts unequivocally derived from the B chromosome in males showing nucleoli attached to the B chromosome at diplotene cells. We designed primers which specifically amplified a 484 bp region of the ITS2 rDNA in the B chromosome, on the basis of an adenine insertion being exclusive of the B_24_ chromosome rDNA [39]. PCR amplification with these primers was negative on gDNA in all 20 B-lacking individuals (10 males and 10 females) analysed. However, it was positive on gDNA from all B-carrying individuals analysed (34 males and 13 females), thus showing the high specificity of this reaction for the B chromosome rDNA. When the same assay was performed on cDNA, no amplification was observed in 0B individuals. In B-carrying individuals, however, the 484 bp fragment was obtained in some individuals, suggesting that the B chromosome rDNA is facultatively active.

In males, we observed a very high correspondence between the molecular and cytological analyses, i.e. the presence of the ITS2_B transcripts, deduced from the PCR amplification of the 484 bp fragment, and the existence of NOR activity in the B chromosomes, deduced from the presence of nucleoli attached to them. Presence of both evidences for B activity was observed in 18 males, and their absence in 13 males (see Table 4). The exceptions were males no. 43, 72 and 74. In the two former, there were ITS2_B transcripts but no nucleoli were found attached to the B chromosomes at diplotene, whereas the reverse situation was found in male no. 74, i.e. PCR experiments failed to show the presence of the ITS2_B transcripts but two diplotene cells, out of the 20 analysed, showed nucleoli attached to B chromosomes. This indicates that both ways for ascertaining B-NOR activity (i.e. cytological and molecular) may fail when the expression level is low. The situation observed in males 43 and 72 could be explained by early disorganization of nucleoli at diplotene, the existence of a threshold for silver impregnation preventing nucleolus visualization when B-NOR expression is low, or else B activity in an organ other than testes, since molecular analyses where performed in the whole body excepting testes. The possibility of differences in B-NOR expression among tissues of a same individual merits future research. The absence of PCR amplification in male 74 might be due to a very low number of ITS2_B transcripts which hampered the final success of transcript detection. Alternatively, the possibility exists that the nucleoli attached to the B chromosomes and the molecular expression of the B rDNA do follow different temporal schedules, since the nucleoli attached to Bs that were observed at pachytene-diplotene were formed during leptotene-zygotene, implying that the B-transcripts might have been produced several days before nucleolus observation, so that it is conceivable that a same male may, at a given time, show nucleoli attached to their B chromosomes at pachytene but not ITS2_B transcripts since its production had finished several days before.

In females, this correspondence cannot be tested since it is not possible to analyse B-NOR expression cytologically. But the close correspondence between cytological and molecular results, observed in males, allow inferring B-NOR expression in females through the PCR assay only. This showed the presence of the 484 bp fragment in 10 out of the 13 B-carrying females, suggesting that the rRNA genes in the B_24_ chromosome are active in most females, whereas it is active in only about half of males.

The active or inactive status of the rDNA in the B chromosome may depend on epigenetic modifications such as DNA methylation and/or histone methylation or acetylation. In *E. plorans*, the NOR activity observed by Cabrero *et al.* [36] in the B_2_ chromosome fused to the longest autosome was later shown to be related with undermethylation of the rDNA [40]. In addition, it has been shown that B chromosomes in this species are hypoacetylated for lysine 9 in the H3 histone [41].

The role of B-derived rRNA transcripts and the mechanism of transcription of the B repeats remain to be elucidated. A possibility is that these transcripts might have structural functions in the organization and regulation of the Bs themselves [42], [43]. But, in *E. plorans*, B chromosomes in the Torrox population might also contribute to the total rRNA demanded by the cell, since the activity of the B-NOR is associated with a decrease in the activity of the NORs in the A chromosomes, so that total cell nucleolar area does not change [37], [38]. Unless the rDNA located in the B chromosome would have preserved functionality, an increase in B-derived rRNA transcripts could lead to an increasing proportion of abnormal rRNA copies that could be detrimental for the fitness of B-carrying individuals. This possibility, however, needs further research to determine the relative amount of the B-rRNA transcripts (in respect to those derived from the A chromosomes) and whether the B-rRNA transcripts are completely functional.

Given that total cell nucleolar area in B-carrying males does not change despite B chromosome contribution to nucleolus formation, Teruel *et al.* suggested that cell regulation of rRNA demands should lead to a decrease in the nucleolar area contributed by the A chromosomes [37], [38]. Therefore, the presence of rDNA in the B chromosome of *E. plorans* seems to have implications for the regulation of rDNA expression and its relationship with the genomic amount of this kind of repetitive DNA. Ide *et al.* have recently suggested that the presence of many copies of rDNA, many of which are transcriptionally inactive, makes genomes less sensitive to DNA damage, because the extra copies facilitate recombinational repair [44]. As shown by these authors, the lower DNA sensivity in yeast strains with high copy number depends on a lower ratio of transcribed rRNA genes than that in strains with low copy number whose rRNA genes are up-regulated. On this basis, the fact that B-NOR activity in *E. plorans* does not change total cell nucleolar area [37], [38] might be the result of a rigid A genome control on rDNA expression, perhaps because excessive activity would decrease individual fitness through a lower protection against DNA-damaging agents. Similar reasons would explain why the grasshopper *Stauroderus scalaris* carries large amounts of rDNA in all chromosomes but only those in chromosome 3 are active [45].

The case of nucleolar expression in the B chromosomes of *E. plorans* resembles nucleolar dominance, a phenomenon usually observed in interspecific hybrids, by which the rDNA clusters from one of the parental species are active whereas those from the other parental species are inactive [46]. Nucleolar dominance seems to be reversible since, in some individuals, the silenced rDNA can be reactivated under certain conditions, e.g. in developmental stages demanding more ribosomal synthesis [47]. In *E. plorans*, the rDNA located in the B_2_ chromosome is usually silenced [48] whereas that in the three smallest autosomes and the X chromosome is active in a variable proportion of diplotene cells in all males [49]. The B_24_ chromosome arose from B_2_ and replaced it in the Torrox population during the 1980's [50], from where it is currently expanding towards the west [51] and the east [52]. In the last years, for unknown reasons, the rDNA in the B_24_ chromosome has been derepressed since, in 1999, it showed NOR activity in 31% of males [37], and this figure increased to about 40% in 2003–2004 [38] and 48.3% in 2008 (this paper, Table 2). Therefore, it seems that the “active B-NOR” phenotype is increasing in frequency in this population. Remarkably, however, the proportion of diplotene cells showing the phenotype in B-carrying males was about the same in 1B, 2B and 3B males, thus showing no odd-even pattern for this trait, in contrast to many B chromosome effects (see [53]).

The B_24_ chromosome thus constitutes excellent material to analyse the regulation of rDNA expression at intragenomic level, and the PCR assay described here could contribute significantly to this task because it permits to analyse B-rDNA expression in every cell type, tissue and organ at every developmental stage where RNA can be isolated. Likewise, it permits to test for B-rDNA expression in other populations harbouring other B variants, which is crucial for understanding the biological role of these B chromosomes. B_24_ showed significant drive in 1992, i.e. when it was finishing the replacement of B_2_ in Torrox [50], but a few years later it had lost drive [54]. The recurrent expression of its rDNA could thus be a new pathway for B_24_ evolution in this species, since it is contributing to an important host function, i.e. rRNA production. It also suggests that B chromosomes are not as genetically inert as was previously thought, and they may even contain some protein-coding genes whose expression can interfere with important ontogenetic processes such as, for instance, sex determination [23].

## Materials and Methods

### Experimental material

A total of 67 males and females of the grasshopper *Eyprepocnemis plorans* were collected in Torrox (Málaga, Spain) in 2003, 2004, 2007 and 2008 (Table 1). For cytological analysis, males and females were anaesthetized before dissecting out testes and ovarioles, respectively, which were then fixed in freshly prepared 3 1 ethanol-acetic acid and stored at 4°C. Ovarioles were immersed in 5% colchicine in insect saline solution for 3 hours prior to fixation. For molecular analysis, body remains were frozen in liquid nitrogen and stored at −80°C prior to DNA and RNA isolation.

### Cytological analysis of B-NOR expression

The number of B chromosomes in each individual was determined in 2% lactopropionic orcein squash preparations. Cytological evidence for the activity of the B chromosome NOR was obtained from the presence of nucleoli attached to B chromosomes in diplotene cells. For this purpose, testis preparations were submitted to the silver impregnation technique [7] following the procedure described in [55]. These preparations were additionally stained with 1% Giemsa to differentiate the chromatin (blue) from the nucleoli (yellow to deep brown) (Figure 1). At least twenty diplotene cells per male were analysed. Cells were photographed with an Olympus digital camera (DP70).

### Molecular analysis of B-NOR expression

A total of 44 males (10 with 0B, 20 with 1B, 6 with 2B, 7 with 3B and 1 with 4B) and 23 females (10 with 0B, 2 with 1B and 11 with 2B) were analyzed at the molecular level. Each individual body was divided into two hemibodies, each of which was used for genomic DNA and total RNA isolation. Genomic DNA extraction was performed using GenElute Mammalian Genomic DNA Miniprep (Sigma), following manufacturer's recommendations.

Total RNA was extracted with Real Total RNA spin plus (Real), following manufacturer's recommendations, except increasing DNase I treatment up to 20 units to ensure complete removal of any possible contaminating genomic DNA. After a second cleaning with DNase I, we assessed the quality of the isolated RNA by electroforesis in a MOPS (3-N-morpholinopropanesulfonic acid) denaturing agarose gel, based on the presence of the 28S and 18S ribosomal RNA bands and the absence of low molecular weight fragments.

For PCR experiments on genomic (gDNA) and complementary (cDNA) DNA, a primer pair (ITSA and ITSB) was designed on the basis of the ITS2 sequences reported by M. Teruel (accession numbers: JN811827–JN811836 for 0B individuals, and JN811886–JN811902 for microdissected B chromosomes), who showed that an adenine insertion was present only in rDNA obtained from microdissected B chromosomes but was absent in rDNA sequences from 0B individuals [39]. We thus anchored the reverse (ITSB) primer in the ITS2 region including this adenine. PCR reaction with the “forward ITSA (5′ TGGAGCCGTACGACGAAGTG 3′)” and “reverse ITSB (5′CGTTGTACGAAAGAGTTTGAG 3′)” primers was adjusted to yield a 484 bp DNA fragment from the desired ITS2 region, only in presence of the inserted adenine in the template (complementary to the underlined T in the ITSB primer). PCR mixture consisted of 200 µM dNTPs, 10 µM each primer, 20 ng genomic DNA and one unit of Taq polymerase (New England, BioLabs) with 1× buffer in a final volume of 25 µl. PCR conditions were the following: an initial denaturation at 94°C for 5 min and 30 cycles of 94°C for 30 s, 62,5°C for 40 s, 72°C for 45 s and final extension of 72°C for 7 min. PCR products were visualized in an electrophoresis 1.5% agarose gel and the amplified fragment was cloned into TOPO TA vector (Invitrogen) and subsequently sequenced in both directions (Macrogen). Searching for sequence homology in databases was performed using BLAST (Basic Local Alignment Search tool) at NCBI site. Alignments were performed with Bioedit software (version 7.0.9.0).

Complementary DNA (cDNA) was synthesized from total RNA with SuperScript III First-Strand Synthesis SuperMix (Invitrogen), following manufacturer's protocol, and it was used as template for PCR experiments with the ITSA and ITSB primers. PCR reactions on cDNA contained 2000 µM dNTPs, 10 µM of each primer, 1U Taq polymerase (New England, BioLabs) and 30 ng cDNA. PCR conditions were as follows: initial denaturation at 94°C for 5 min, 30 cycles of 94°C for 30 s, 62,5°C (males) and 62,7°C (females) for 40 s, 72°C for 45 s and a final extension of 72°C for 7 min. Analysis of PCR products, cloning of amplified fragments, DNA sequencing and homology search in the databases were performed as for genomic DNA (see above).

## Supporting Information

Figure S1 Nucleotide sequence of the DNA amplified with the ITS2A and ITS2B primers.
(DOC)
