# Supplementary material for: B-Chromosome Ribosomal DNA Is Functional in the Grasshopper Eyprepocnemis plorans
Source: PLoS One. 2012 May 3;7(5):e36600. doi: 10.1371/journal.pone.0036600 (PMC3343036; doi:10.1371/journal.pone.0036600)
Supplement: Figure S1 — Nucleotide sequence of the DNA amplified with the ITS2A and ITS2B primers. (DOC) [file pone.0036600.s001.doc]

1 TGGAGCCGTACGACGAAGTGGCGGCGGTTTGTGCTTGCACGACGCCGGCCGCCACACACA

61 TTTGGAACAGGGCCTGTCAAAGGGCCCAGTCCCGCCTATGCAACAGCAGGCTTTGCCTGA

121 CAAGCAAATGTATGAAAAAAGATCACCCAGGACGGTGGATCACTCGGCTCGTGGGTCGAT

181 GAAGAACGCAGCAAATTGCGCGTCGACATGTGAACTGCAGGACACATGAACATCGACGTT

241 TCGAACGCACATTGCGGTCCATGGATTCCGTTCCCGGGCCACGTCTGGCTGAGGGTCGGC

301 TACGTATACTGAAGCGCCAAGGCGTTTCGGAGACTTGGGAGCGTCGTGGTACGCCCGTCG

361 TGCCGCGTCTCCTCAAATGTGGAGTGCGCGCCCGTCGCTCGGGCGGTTCGCATACCGGTA

421 CTGTGTCTCGGTAGCGTGCACAGCTGCCCGGCGGTGCGGCGCGCTCAAACTCTTTCGTAC

481 AACG
